# Physiological traits determining high adaptation potential of sweet briar (*Rosa rubiginosa* L.) at early stage of growth to dry lands

**DOI:** 10.1038/s41598-019-56060-3

**Published:** 2019-12-18

**Authors:** Joanna Gadzinowska, Agnieszka Ostrowska, Katarzyna Hura, Michał Dziurka, Bożena Pawłowska, Tomasz Hura

**Affiliations:** 1 Polish Academy of Sciences, The Franciszek Górski Institute of Plant Physiology, Niezapominajek 21, Kraków, 30-239 Poland; 2 Department of Plant Breeding, Physiology and Seed Science, Faculty of Agriculture and Economics, University of Agriculture, Podłużna 3, Kraków, 30-239 Poland; 3 Department of Ornamental Plants and Garden Arts, Faculty of Biotechnology and Horticulture, University of Agriculture, 29 Listopada 54, Kraków, 31-425 Poland

**Keywords:** Drought, Plant physiology

## Abstract

Little is known about mechanisms of sweet briar adaptation to dry habitats. The species is highly invasive and displaces native plants from dry lands of the southern hemisphere. This study evaluates physiological basis of *Rosa rubiginosa* L. adaptation to soil drought. We performed a pot soil drought experiment and assessed water relations, water use efficiency, gas exchange and photosynthetic apparatus activity. The study also measured the content of chlorophyll, soluble carbohydrates and proline and analyzed plant biomass growth. We hypothesized that the drought stress induced an effective mechanism enabling adaptation of young sweet briar roses to soil water deficit. The study identified several adaptation mechanisms of *R. rubiginosa* allowing the plant to survive soil drought. These included limiting transpiration and stomatal conductance, increasing the level of soluble sugars, reducing chlorophyll content, accumulating CO_2_ in intercellular spaces, and increasing the quantum yield of electron transport from Q_A−_ to the PSI end electron acceptors. As a result, young sweet briar roses limited water loss and photoinhibition damage to the photosynthetic apparatus, which translated into consumption of soluble sugars for growth purposes. This study showed that photosynthesis optimization and increased activity of the photosynthetic apparatus made it possible to avoid photoinhibition and to effectively use water and sugars to maintain growth during water stress. This mechanism is probably responsible for the invasive nature of *R. rubiginosa* and its huge potential to displace native plant species from dry habitats of the southern hemisphere.

## Introduction

*Rosa rubiginosa* L., known also as sweet briar, belongs to the group of wild growing roses and is a species native to the northern hemisphere. It was spread, naturally or through anthropogenic and animal participation into the southern hemisphere^1–3^. Sweet briar rose was introduced to South America, Australia and New Zealand, where it adapted to the new environment and occupied such areas as forests, and dry lands such as steppes. In South America, its wild growing shrubs can be found predominantly in Chile and Argentina^2,4^. It is a fast-growing shrub with a bushy and effusing habit that prefers habitats exposed to sunlight but may also grow in partial shade^5^.

In many regions of the southern hemisphere sweet briar rose is treated as a highly expansive weed. Abundance of herbivorous fauna in the southern hemisphere, for which the fruits of sweet briar are valuable forage, is an additional factor of this species propensity to expansion^6^. Plant achenes undergo scarification in the animal digestive tract, which after their defecation accelerates the seed germination. From the ecological perspective, invasive varieties of sweet briar rose may pose a threat to the ecosystem they grow in since they contribute to the displacement and extinction of valuable species of indigenous plants.

*R. rubiginosa* was introduced to New Zealand in 1800 and has since been treated as a truly parasitic species^7^. In New South Wales (Australia), since 1919, it has been treated as one of 20 most persistent weeds^8^. In 1960, New Zealand tried to apply biological methods to control invasive varieties of sweet briar rose. To reduce the population of this shrub, an attempt was made to use rose-seed megastigmus, *Megastigmus aculeatus* (of hymenoptera order) but only about 8% of the rose seed were infested and destroyed. There were also plans to use another insect, i.e. rose bedeguar gall, *Diplolepis rosae* (also of hymenoptera order) to control the growth of this rose but the project was withdrawn^9^.

Sweet briar rose is tolerant to soil drought conditions prevailing in urban habitats, pollution, poorer soils, frosts and diseases^10–15^. Its resistance to adverse environmental conditions makes *R. rubiginosa* suitable for widespread applications in, *inter alia*, naturalistic urban green areas or for the reclamation of polluted urban soils^16,17^. However, there is no information in the literature on the physiological and biochemical mechanisms of sweet briar rose adaptation to adverse environmental factors, including soil drought. So far, the only paper that investigated this topic was published in 1999 and focused on the analysis of photosynthetic activity conducted on leaves cut (drought simulation) from sweet briar rose^18^. However, some information on the ecology of this species may be found in the literature^2,3,15^.

Therefore, identification of physiological and biochemical adaptation mechanisms to dry environments may help develop effective methods to reduce the uncontrolled spread of this species. In this study, we investigated the response of young, one year old plants with already woody shoots, to soil water deficit. We assumed that the photosynthetic apparatus plays a key role in the mechanisms of adaptation to soil drought. Its activity is indispensable for the synthesis of sugars involved in osmoregulation and water retention in the cells. To verify this hypothesis, a pot experiment in a garden tunnel was conducted, in which we analyzed water relations, photosynthetic activity, proline content and plant biomass growth.

## Results

**Figure 1:**
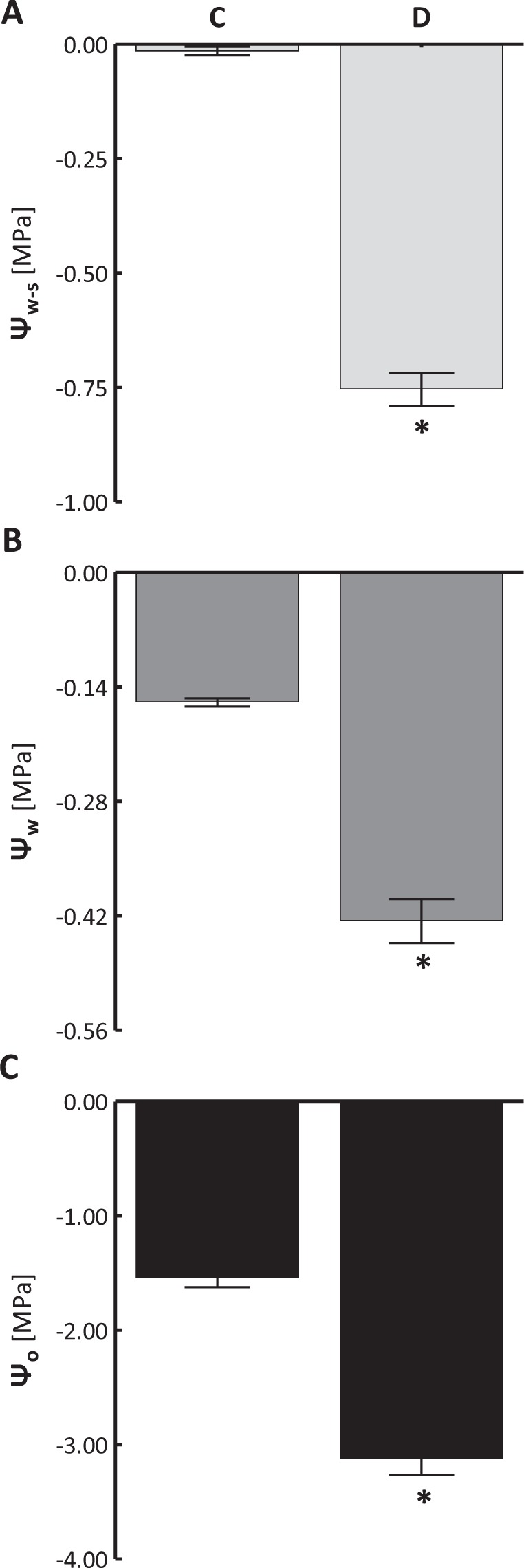
Changes in soil water potential (**A**, Ψ_W-S_), leaf water potential (**B**, Ψ_W_) and osmotic potential (**C**, Ψ_O_) in sweet briar on the thirtieth day of soil drought. C – control, D – drought. Mean values ± SE (n = 10 for Ψ_W-S_; n = 9 for Ψ_W_ and Ψ_O_). Asterisks mark differences significant at p < 0.05 vs. control, Student’s t-test.

On the thirtieth day of drought (D), soil water potential (Ψ_W-S_) reached ca. −0.75 MPa and was significantly lower than in the optimally watered variant (−0.006 MPa) (C) (Fig. 1A). Soil water deficit induced water stress in the leaves of sweet briar manifested in reduced water potential (for C: Ψ_W_ = −0.16 MPa, for D: Ψ_W_ = −0.43 MPa) (Fig. 1B) and osmotic potential (for C: Ψ_O_ = −0.54 MPa, for D: Ψ_O_ = −3.12 MPa), as compared with regularly irrigated plants (Fig. 1C).

**Figure 2:**
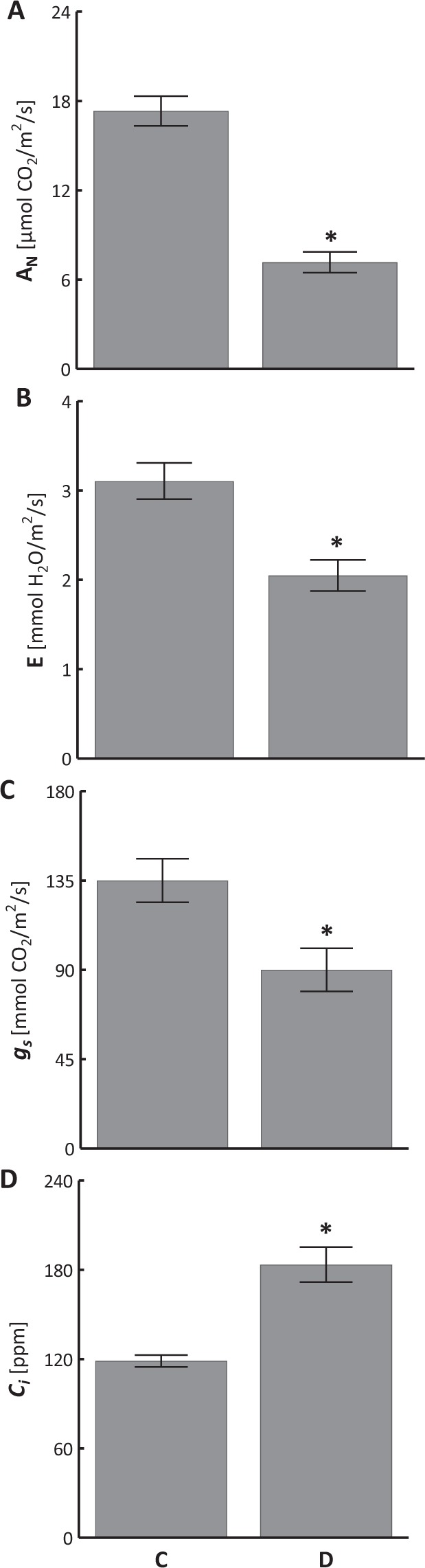
Changes in photosynthesis rate (**A**, A_N_), transpiration rate (**B**,**E**), stomatal conductance (**C**, *g*_*S*_), and intercellular concentration of CO_2_ (**D**, *C*_*i*_) in sweet briar on thirtieth day of soil drought. C – control, D – drought. Mean values ± SE (n = 10). Asterisks mark differences significant at p < 0.05 vs. control, Student’s t-test.

Leaf dehydration caused significant reduction of photosynthesis (A_N_) (Fig. 2A) and transpiration rate (E) (Fig. 2B), as well as stomatal conductance (*g*_*S*_) (Fig. 2C). On the thirtieth day of water deficit, a clear increase in intercellular concentration of CO_2_ (*C*_*i*_) was noted (Fig. 2D).

**Figure 3:**
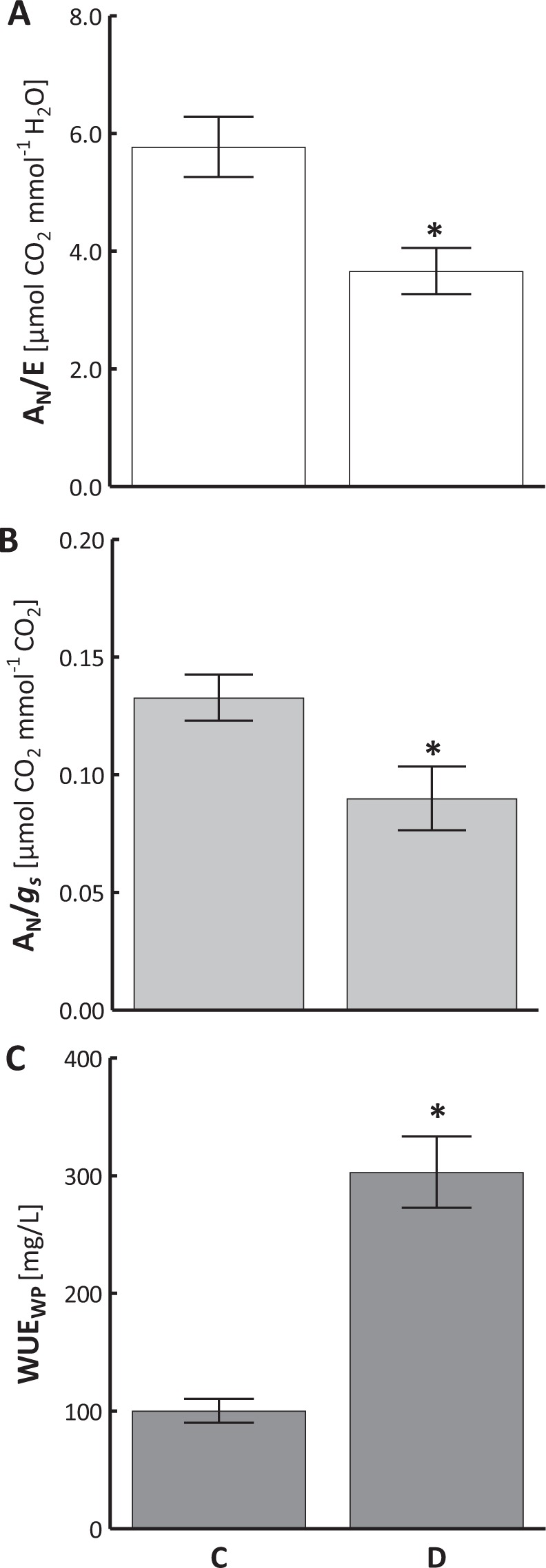
Changes in water use efficiency calculated based on photosynthesis rate to transpiration rate ratio (**A**, A_N_/E) and photosynthesis rate to stomatal conductance ratio (**B**, A_N_/*g*_*S*_), and the ratio of biomass growth to the amount of provided water (**C**, WUE_WP_) in sweet briar on thirtieth day of soil drought. C – control, D – drought. Mean values ± SE (n = 10 for A_N_/E and A_N_/*g*_*S*_; n = 15 for WUE_WP_). Asterisks mark differences significant at p < 0.05 vs. control, Student’s t-test.

At the leaf level, we saw a significant reduction in water use efficiency (WUE), calculated as a ratio of photosynthesis rate and transpiration rate, i.e. so called instantaneous WUE (Fig. 3A). A similar drop was confirmed for so called intrinsic WUE, i.e. a ratio of A_N_ to stomatal conductance (Fig. 3B). However, at the whole plant level WUE values increased during soil drought, as showed in the ratio of plant biomass growth and the amount of water provided (Fig. 3C).

**Figure 4:**
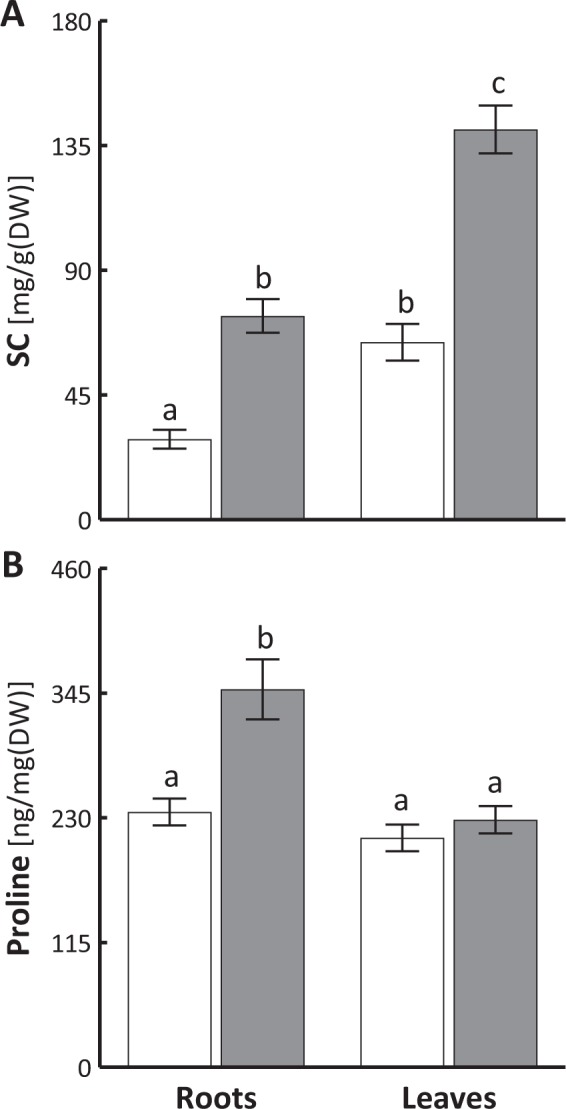
Changes in the content of soluble carbohydrates (**A**, SC) and proline (**B**) in the leaves and roots of sweet briar on thirtieth day of soil drought. C – control, D – drought. Mean values ± SE (n = 5). Duncan’s test at 0.05 probability level was performed in order to determine the significance of differences between treatment means. Means indicated with the same letters are not significantly different.

Drought enhanced the levels of soluble carbohydrates (SC) both in the roots and leaves of sweet briar (Fig. 4A). Their levels in the leaves were higher than in the roots for both control and drought conditions. After 30 days of drought, only roots exhibited a significant increase in proline content (Fig. 4B). In dehydrated leaves, its level was similar as in those optimally hydrated.

**Figure 5:**
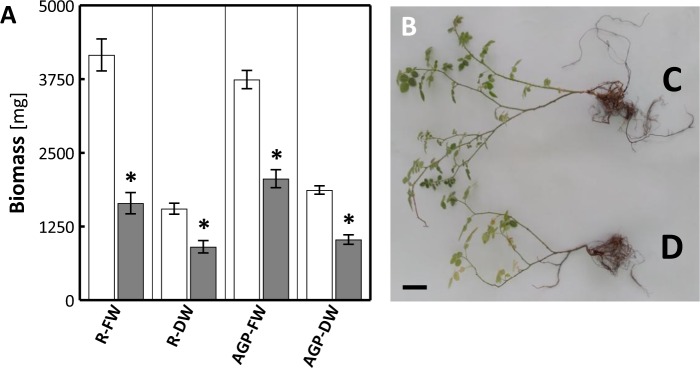
(**A**) Changes in fresh (FW) and dry weight (DW) of above ground parts (AGP) and roots (R) of sweet briar on thirtieth day of soil drought. White bars – control, gray bars – drought. Mean values ± SE (n = 10). Asterisks denote significant differences in R-FW, R-DW, AGP-FW and AGP-DW for p < 0.05 vs. control, Student’s t-test. (**B**) Changes in plant height. C – control, D – drought. Bar = 5 cm.

Limited water content determined for the thirtieth day of the study considerably reduced fresh (FW) and dry weight (DW) of above ground parts and roots (Fig. 5A). Soil drought limited also elongation growth of sweet briar, clearly manifested in lower plant height (Fig. 5B). Also, the root system of drought exposed plants was much less developed than in controls (Fig. 5B).

On the thirtieth day of drought the photosynthetic apparatus of sweet briar showed higher activity than in optimally hydrated plants (Table 1). Significant changes occurred for a majority of the analyzed parameters of chlorophyll fluorescence except for ABS/CS_m_ that involves the amount of energy absorbed by antennas (1922 for control, 1977 for drought, 103.9% of control).

**Table 1:** Changes in 10 chlorophyll fluorescence parameters and chlorophyll (Chl) content on the thirtieth day of soil drought. C – control, D – drought, % of C – D values exhibited as percentage of control. Mean values ± SE (n = 10). Asterisks at individual parameters mark differences significant at p < 0.05 vs. control, Student’s t-test.

Parameters	C	D	% of C
F_v_/F_m_	0.727 ± 0.008	0.771 ± 0.005*	106.0
ABS/CS_m_	1922 ± 39	1997 ± 36	103.9
TR_o_/CS_m_	1399 ± 34	1541 ± 36*	110.2
ET_o_/CS_m_	380 ± 21	506 ± 34*	133.2
DI_o_/CS_m_	524 ± 17	456 ± 8*	87.0
RC/CS_m_	511 ± 15	603 ± 24*	118.0
PI	0.274 ± 0.026	0.510 ± 0.054*	186.1
δR_o_	1.59 ± 0.06	2.27 ± 0.11*	142.8
φR_o_	0.098 ± 0.007	0.145 ± 0.004*	148.0
ψR_o_	0.379 ± 0.013	0.443 ± 0.009*	116.9
Chl	4.23 ± 0.24	2.56 ± 0.18*	60.5

Chlorophyll fluorescence parameters recorded during water stress indicated enhanced efficiency of excitation energy use (reduction in DI_o_/CS_m_ – 87% of control, increase in ET_o_/CS_m_ – 133% of control, increase in TR_o_/CS_m_ – 110% of control). Chlorophyll fluorescence measurements revealed also a rise in quantum yield of electron transport (φR_o_, 148% of control) from Q_A_^−^ to the end acceptors in PSI. The changes in the photosynthetic activity recorded on the thirtieth day of drought were accompanied by significant drop in chlorophyll content (Table 1).

## Discussion

During their evolution, plants have developed adaptation mechanisms to habitats of low soil water content^19,20^. Those plants include species with high potential for colonization of dry sites. One of these is *R. rubiginosa*, the invasive populations of which conquered dry areas of the southern hemisphere^3^. Its high ability to thrive at unfavorable soil moisture level may be due to developing specific and effective adaptation mechanisms. This is particularly important during interaction of young plants of low biomass (one year old plants in our experiment) with intense stress represented here by soil drought (in our experiment involving 30 days of limited irrigation).

Common responses to leaf dehydration include reduction of photosynthesis, transpiration and partial closure of stomata^21,22^. These are adaptive responses aimed at limiting water loss but they also inhibit plant growth^23,24^.

Sweet briar is a C_3_ plant^25^. In our pot experiment, limited watering induced water stress (Fig. 1B), manifested in reduced photosynthetic activity (Fig. 2A), limited transpiration (Fig. 2B) and partial closing of the stomata (Fig. 2C). Despite limited gas exchange *R. rubiginosa* still accumulated CO_2_ (*C*_*i*_) in its intercellular spaces (Fig. 2D). Enhanced *C*_*i*_ occurs mainly in plants with CAM photosynthesis type^26^. At limited stomatal conductance an increase in CO_2_ concentration may be an alternative for photosynthesis optimization in the investigated species during leaf dehydration. Studies on water stress in C_3_ plants usually indicate lowering of intercellular concentration of CO_2_^27–29^. The accumulation of CO_2_ by *R. rubiginosa* makes the plant somewhat independent of atmospheric CO_2_, the availability of which is limited by partial close of the stomata. Another reason may involve changes in the internal leaf structure caused by cell dehydration and shrinking. Hura *et al*.^30^ showed that leaf dehydration in sweet briar exposed to salt stress causes cell shrinking and widens intercellular space available for CO_2_ accumulation.

High *C*_*i*_ values were accompanied by intensified activity of the photosynthetic apparatus and reduced chlorophyll content (Table 1). Lowering chlorophyll content under water stress is one of the ways to avoid photoinhibition damage to the photosynthetic apparatus^31–34^. Kyparissis *et al*.^35^ found that summer survival of *Phlomis fruticosa* shrub under Mediterranean climate depended on avoidance of photoinhibition damage through decreasing chlorophyll content. Water stress makes plants more susceptible to damage inflicted by excessive UV-Vis radiation^36^.

In our opinion, the increase of intercellular CO_2_ in dehydrated plants is a means for photoinhibition prevention by providing a sink for electron transport. This statement seems supported by high values of φR_o_ (Table 1) indicating increase of the quantum yield of electron transport from Q_A−_ to the PSI end electron acceptors^37^ and NADPH_2_ generation that together with ATP is used in the dark reaction to reduce CO_2_ to carbohydrates^38^. The relationship between high values of *C*_*i*_ and photoprotection in *R. rubiginosa* may be concluded from a significant increase in F_v_/F_m_ ratio (Table 1) representing the photosystem performance. The value of PI increased significantly under drought together with an increase in intercellular CO_2_. This, accompanied by the decrease in DI_o_/CS_m_, suggests that higher values of *C*_*i*_ in drought treated plants guarantee a sufficient sink for electrons, thereby decreasing the need for energy dissipation (low values of DI_o_/CS_m_) (Table 1).

Increased activity of the photosynthetic apparatus and photosynthesis optimization under leaf dehydration enhanced the content of soluble carbohydrates in both roots and leaves (Fig. 4A). Soluble carbohydrates may be utilized during plant growth^39^, but as osmotically active substances they also retain water in the cell and limit its dehydration^40^. Proline is another substance with osmoregulatory function but in our study its increased accumulation in the roots (Fig. 4B) is probably associated with its signaling role in the expression of drought resistance genes^41^.

## Conclusions

**Figure 6:**
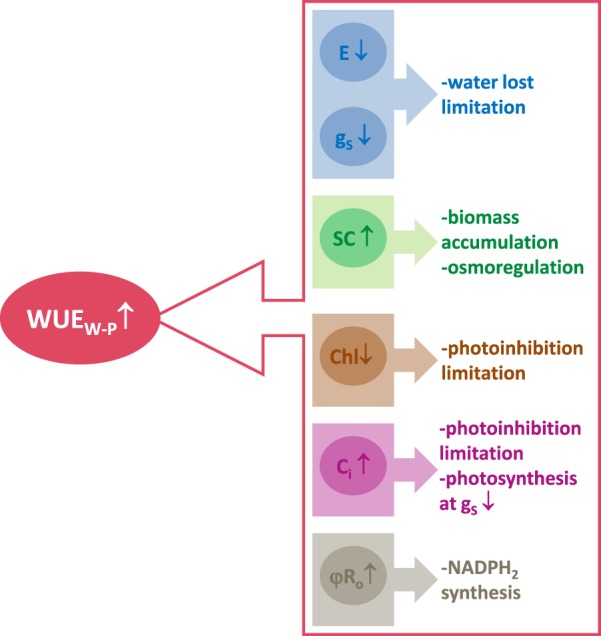
Physiological and biochemical mechanisms of *Rosa rubiginosa* adaptation to soil water deficit that allow for effective water management at the entire plant level (WUE_WP_). E - transpiration rate, *g*_*s*_ - stomatal conductance, SC - soluble carbohydrates, Chl - chlorophyll content, *C*_*i*_ – intercellular concentration of CO_2_, φR_o_ - quantum yield of electron transport from Q_A−_ to the PSI end electron acceptors. ↑- significant increase of a parameter value, ↓- significant decrease of a parameter value.

Sweet briar drought adaptation mechanisms outlined above include efficient water managements at the entire plant level (WUE_WP_) (Fig. 3C). Considering the study outcomes, we suggest that *R. rubiginosa* response to soil drought involves the adaptation mechanisms resulting not only in water deficit tolerance but also ensuring effective water management (Fig. 6). These mechanisms include limiting transpiration and stomatal conductance, increasing the level of soluble sugars, reducing chlorophyll content, accumulating CO_2_ in intercellular spaces, and increasing the quantum yield of electron transport from Q_A−_ to the PSI end electron acceptors. As a result, sweet briar exposed to drought stress is capable of limiting both water loss and photoinhibition damage to the photosynthetic apparatus.

Moreover, photosynthesis optimization during water stress triggered consumption of soluble carbohydrates for growth processes. This should be attributed to huge invasive potential of *R. rubiginosa*, whose growth dynamics in dry environment needs to be competitive to native species of the southern hemisphere.

## Material and Methods

### Plants material and growth conditions

The study involved 13 month old sweet briar roses after their first overwintering, and with fully lignified stems. After stratification, the germinating seeds were sown into pots (1 L, one plant per pot) filled with a mixture of Klasmann-Deilmann TS1 substrate and sand (v/v: 1:2). The seeds (collected in the autumn 2016) came from a shrub wildly growing at a site in Pychowice near Kraków, Poland (50°02′01.2″N, 19°51′58.6″E).

From the beginning of May 2018 to the end of June 2018 the plants grew under a garden tunnel equipped with sensors monitoring temperature changes. Over 30 days of the experiment, minimum temperature in the tunnel varied from 8.9 °C to 17.5 °C, maximum temperature from 14.1 °C to 30.8 °C, and average temperature from 13.2 °C to 23.4 °C (Supplementary Information). Photosynthetic photon flux density (PPFD) in the tunnel was monitored with a QSPAR Quantum Sensor (Hansatech Instruments LTD, Kings Lynn, England) between 11.00 a.m. and 12 p.m. PPFD ranged from 19–36 µmol photons m^−2^s^−1^ (six cloudy and rainy days) to 142–348 µmol photons m^−2^s^−1^ (24 sunny or mostly sunny days) (Supplementary Information). Prior to the experiment, all plants were everyday provided with the same amount of water depending on air temperature (from 100 to 400 ml).

### Drought conditions

Soil drought was applied by limiting watering for 30 days – from 8 May to 6 June 2018. Optimally watered plants received in total 9 L of water per pot, while those exposed to drought only 2.7 L, i.e. 70% less. Irrigation frequency and the amount of water depended on the temperature inside the garden tunnel. For the last three days (28–30) of soil drought the plants did not receive any water.

### Measurements

The measurements were conducted on the thirtieth day of soil drought. Quantitative analysis involved the first fully developed top leaf (each leaf developed seven leaflets). Chlorophyll fluorescence and chlorophyll content were determined for the top leaflet of the largest assimilation area.

#### Soil water potential (Ψ_w-s_), leaf water potential (Ψ_w_), leaf osmotic potential (Ψ_o_)

The measurements were performed with a psychrometer HR 33 T (WESCOR, Inc., Logan, Utah, USA), equipped with leaf sample chambers C-52 (WESCOR, Inc., Logan, Utah, USA)^37^. To assess Ψ_w-s_, we collected soil samples of equal amount from the root zone and placed them in C-52 chambers for 60 min. Water potential was also evaluated in leaf discs. Five leaf discs, 5 mm in diameter, collected from each leaflet, were placed in C-52 chambers and left for 60 minutes. Leaf sap used in the analysis of the osmotic potential was extracted with a syringe. Filter paper discs soaked in the sap were placed in the chambers and left for 40 minutes. All measurements were taken in the dew point mode.

#### Gas exchange

Photosynthesis rate (A_N_), transpiration rate (E), stomatal conductance (*g*_*S*_), and intercellular concentration of CO_2_ (*C*_*i*_) were measured using an infrared gas analyzer LCpro-SD (ADC BioScientific Ltd., UK). The conditions in the measurement chamber were as follows: carbon dioxide concentration 360 µmol CO_2_ mol^−1^ air, air humidity equal to ambient humidity, temperature 28 °C, PAR intensity 600 µmol photons m^−2^s^−1^. The measurements were carried out between 10:00 a.m. and 12:00 p.m. and involved the first three leaflets of the largest assimilation area.

#### Water use efficiency

Additionally, we determined an instantaneous water use efficiency index (WUE) representing A_N_/E ratio, and intrinsic WUE representing A_N_/*g*_*S*_ ratio. WUE of the whole plant (WP) was estimated as follows: WUE_WP_ [g/L] = (FPB − IPB)/TWC, where FPB – final plant biomass, IPB – initial plant biomass, TWC – total water consumption.

#### Chlorophyll fluorescence measurements

Measurements were carried out with a fluorometer Handy PEA (Hansatech Ltd. Kings Lynn, UK) after 25 min of leaf adaptation to darkness. Quantum yield of PSII (F_v_/F_m_) was calculated according to van Kooten and Snel^42^. The excitation irradiance was 3000 μmol (quanta) m^−2^ s^−1^ (650 nm). The following parameters were calculated per excited leaf cross-section (CS_m_): ABS/CS_m_ (energy absorption by antennas), PI (overall performance index of PSII photochemistry), DI_o_/CS_m_ (energy amount dissipated from PSII), RC/CS_m_ (number of active reaction centers), ET_o_/CS_m_ (amount of energy used for the electron transport), TR_o_/CS_m_ (amount of excitation energy trapped in PSII reaction centers), ψR_o_ (probability, at time 0, that a trapped exciton moves an electron into the electron transport chain beyond Q_A−_), δR_o_ (the efficiency with which an electron can move from the reduced intersystem of electron acceptors to the PSI end electron acceptors), φR_o_ (the quantum yield of electron transport from Q_A−_ to the PSI end electron acceptors). The parameter calculation was based on the theory of energy flow in PSII using the JIP test^43,44^. The measurements involved the top leaflet of the largest area.

#### Chlorophyll content

Chlorophyll content in the fully expanded leaves was measured with a chlorophyll meter (Chlorophyll Content Meter, CL-01, Hansatech Instruments Ltd., UK). The measurements involved the top leaflet of the largest area.

#### Soluble carbohydrate (SC) content

Total soluble carbohydrate (SC) content was determined with anthrone (dissolved in concentrated sulfuric acid) added to aqueous extracts of leaf samples and incubated for 15 min at 90 °C. Absorbance was measured spectrophotometrically (Ultrospec 2100 Pro, Amersham Biosciences, Cambridge, UK) at 620 nm. Glucose was used to prepare a calibration curve.

#### Proline content

Proline content was measured spectrophotometrically according to Hura *et al*.^45^. Briefly, 5 mg of lyophilized and powdered samples were extracted by 5 min in 0.5 ml of 3% 5-sulfosalicylic acid. Extracts were centrifuged at 21 000 g for 15 min. Then, 200 μl of the supernatant were transferred into screw cap vials and 200 μl of concentrated formic acid and 400 μl 3% ninhydrin reagent in 2-methoxyethanol were added. The samples were heated for 30 min at 95 °C in a water bath, and then transferred into 96-well plates. Absorbance was measured at 514 nm with a micro-plate reader (BioTek, USA).

#### Plant biomass

Above ground parts of plants were weighted immediately after sampling (fresh weight, FW). Then, after an oven incubation at 80 °C for 78 h the samples were reweighed (dry weight, DW).

### Statistical analysis

Statistical analysis was carried out using Statistica v. 12 (Statsoft Inc., Tulsa, OK, USA). Analysis of variance was used to determine the main effects of drought stress on physiological and biochemical parameters. Before ANOVA, data were checked for normality and homogeneity of variance. The Duncan’s multiple range test at the probability level of 0.05 was performed to estimate the significance of differences between treatment means. Differences between two means were compared by the Student’s t-test.

## Supplementary information

Supplementary Information
