# Supplementary material for: Physiological traits determining high adaptation potential of sweet briar (Rosa rubiginosa L.) at early stage of growth to dry lands
Source: Sci Rep. 2019 Dec 18;9:19390. doi: 10.1038/s41598-019-56060-3 (PMC6920414; doi:10.1038/s41598-019-56060-3)
Supplement: Supplementary file 1 — Supplementary Information [file 41598_2019_56060_MOESM1_ESM.pdf]

Physiological traits determining high adaptation potential of sweet briar (Rosa rubiginosa L.) at early stage of growth to dry lands

Joanna Gadzinowska, Agnieszka Ostrowska, Katarzyna Hura, Michał Dziurka, Bożena Pawłowska, Tomasz Hura

Supplementary Information

From the beginning of May 2018 to the end of June 2018 the plants grew under a garden tunnel equipped with sensors monitoring temperature changes. Over 30 days of the experiment, minimum temperature in the tunnel varied from 8.9ºC to 17.5ºC, maximum temperature from 14.1ºC to 30.8ºC, and average temperature from 13.2ºC to 23.4ºC.

Time	Tmin	Tmax	Tavg	PPFD
2018-05-08	12.4	20.6	16.5	163
2018-05-09	13.8	25.8	19.2	211
2018-05-10	14.3	26.3	20.6	173
2018-05-11	12.8	27.5	19.2	203
2018-05-12	13.3	24	19.0	180
2018-05-13	11.8	24.5	18.7	31
2018-05-14	11.1	18.2	14.8	26
2018-05-15	9.2	16.5	13.3	24
2018-05-16	9.7	15.8	13.2	21
2018-05-17	12.5	14.1	13.3	19
2018-05-18	12.9	14.1	13.5	36
2018-05-19	12.3	20.7	15.6	148
2018-05-20	9.7	20.8	15.8	152
2018-05-21	8.9	20.6	15.8	198
2018-05-22	11.1	26.1	18.9	271
2018-05-23	13.6	24.8	18.9	156
2018-05-24	15.4	24.3	19.7	250
2018-05-25	13.6	24.9	19.7	142
2018-05-26	13.6	27.1	20.3	236
2018-05-27	15.4	27.7	22.1	244
2018-05-28	17.1	27.8	22.6	196
2018-05-29	15	29.7	21.9	279
2018-05-30	15.3	28.4	22.3	210
2018-05-31	16.9	30	23.4	348
2018-06-01	15.8	30.8	23.2	332
2018-06-02	17.1	25.5	20.3	210
2018-06-03	17.5	25	20.1	200
2018-06-04	16.1	26.6	21.3	188
2018-06-05	15.9	25.9	21.3	143
2018-06-06	13.2	21.3	17.2	156
